# Osteoporosis Treatment Gap — A Systematic Literature Review

**DOI:** 10.2478/sjph-2026-0022

**Published:** 2026-09-01

**Authors:** Kaja Gril Rogina, Luka Petravič, Christos Oikonomidis

**Affiliations:** National Institute of Public Health - Regional Unit Maribor, Ljubljanska ulica 4, 2000 Maribor, Slovenia; University of Ljubljana, Faculty of Medicine, Vrazov trg 2, 1000 Ljubljana, Slovenia; University Medical Center Maribor, Ljubljanska ulica 5, 2000 Maribor, Slovenia

**Keywords:** Osteoporosis, Fragility fractures, Healthcare disparities, Treatment gap, Care gap, osteoporoza, osteoporotični zlomi, vrzeli v zdravljenju, neenakosti v obravnavi

## Abstract

**Introduction:**

Osteoporosis is a common metabolic bone disease that increases fracture risk, particularly among older adults, and imposes a substantial global burden. Despite advances in risk assessment and treatment, a large treatment gap remains, with many high-risk individuals not receiving care.

**Methods:**

We conducted a systematic review of English-language studies published up to April 2025, identified through searches in multiple databases (PubMed, Scopus, Web of Science, ScienceDirect, Europe PMC). 43 studies were included. Due to the heterogeneity of study designs and treatment gap definitions, a narrative thematic synthesis was performed.

**Results:**

Reported treatment gap estimates varied widely (38.8–94.6%) across countries and study populations. Lower treatment rates were associated with patient factors (male sex, lower socio-economic status, comorbidities) and healthcare system factors (limited physician awareness, institutional barriers, poor interprofessional communication). Higher treatment rates were associated with older age, family history of osteoporosis, prior diagnosis or bone mineral density testing, and participation in structured care programmes.

**Conclusions:**

Substantial global treatment gaps in osteoporosis care persist, particularly following fragility fractures. These gaps arise from multiple patient, physician, and system-level factors, with persistent disparities by sex, ethnicity, comorbidities, and socio-economic status. Findings highlight the need for standardised definitions of the treatment gap and suggest areas for future research and intervention development.

## Izvleček

**Uvod:**

Osteoporoza je pogosta presnovna bolezen kosti, ki povečuje tveganje za zlome, zlasti pri starejših odraslih, in predstavlja pomembno globalno zdravstveno breme. Kljub napredku pri oceni tveganja za zlome in razpoložljivosti učinkovitih možnosti zdravljenja, vrzel v zdravljenju osteoporoze vztraja, saj številni posamezniki z visokim tveganjem, predvsem starostniki, ne prejemajo ustrezne oskrbe. Namen tega sistematičnega pregleda je kategorizirati vzroke za vrzel v zdravljenju osteoporoze.

**Metode:**

Opravili smo sistematični pregled študij v angleškem jeziku, objavljenih do aprila 2025 v podatkovnih bazah PubMed, Scopus, Web of Science, ScienceDirect, Europe PMC. V analizo je bilo vključenih 43 študij. Zaradi heterogenosti zasnove študij in definicij vrzeli je bila izvedena narativna tematska sinteza.

**Rezultati:**

Ocene vrzeli v zdravljenju se med državami in okolji gibljejo med 38,8 % in 94,6 %. Dejavniki, povezani z nižjimi stopnjami zdravljenja, vključujejo moški spol, nižji socialno-ekonomski status, določene pridružene bolezni, pa tudi dejavnike, povezane z zdravstvenim sistemom, kot so pomanjkanje ozaveščenosti pri zdravnikih, institucionalne ovire ter nezadostno medpoklicno sodelovanje. Višje stopnje zdravljenja so povezane s starostjo, družinsko anamnezo osteoporoze, predhodno diagnozo ali meritvijo kostne gostote ter učinkovito usklajeno oskrbo v okviru specializiranih programov.

**Zaključki:**

Kljub učinkovitim diagnostičnim in terapevtskim možnostim po vsem svetu, vrzel v zdravljenju osteoporoze vztraja, zlasti pri ljudeh po zlomu. Ta vrzel izvira iz dejavnikov na ravni bolnika, zdravstvenih delavcev in zdravstvenega sistema ter se kaže v doslednih razlikah glede spola, etnične pripadnosti, pridruženih bolezni in socialno-ekonomskega položaja. Reševanje teh izzivov bo zahtevalo integrirane poti oskrbe, izboljšano klinično izobraževanje in razvoj modelov za usklajeno obravnavo po zlomu.

## 1 INTRODUCTION

Osteoporosis is the most prevalent metabolic bone disease, characterised by decreased bone mass and microarchitectural deterioration of bone tissue, which increases fracture risk (1). Approximately one in three women and one in six men will experience an osteoporotic fracture during their lifetime. In the European Union, more than 23 million individuals are at high risk of such fractures (1, 2). Globally, 178 million new fractures occurred in 2019, representing a 33.4% increase since 1990 and accounting for 25.8 million years lived with disability (YLDs), a 65.3% increase over the same period (2).

Osteoporotic fractures predominantly affect older adults, particularly women, and typically result from low-energy trauma (2, 3). The most common sites include the hip, vertebrae, distal radius, and proximal humerus. These fractures are associated with chronic pain, functional decline, loss of independence, and reduced quality of life (3). Hip fractures in particular frequently require hospitalisation and are associated with complications such as thrombosis (27%), urinary tract infections (12–61%), and pneumonia (7%) (4). Osteoporosis is diagnosed based on low bone mineral density measured by DXA or the presence of a fragility fracture, particularly at the hip or vertebra. Treatment decisions are additionally informed by fracture risk assessment tools such as FRAX (1, 4).

Despite advances in fracture risk assessment, preventive strategies, and clinical guidelines, a large proportion of individuals at high risk remain untreated (1, 5). The SCOPE 21 report estimates an average treatment gap of 71% across Europe among women at high fracture risk (predominantly postmenopausal), ranging from 87% in Bulgaria to 32% in Ireland; in Slovenia, it is approximately 57% (1). The treatment gap is widest among older adults, particularly those in long-term care (5). This gap contrasts with other areas of secondary prevention, such as post–myocardial infarction care, where treatment rates have improved substantially over time (6). Reducing the osteoporosis treatment gap is essential, given its substantial contribution to fracture burden and associated healthcare costs (1).

This systematic review aims to identify and categorise the reported reasons for the osteoporosis treatment gap, intending to inform future interventions to address this issue.

## 2 METHODOLOGY

This review was conducted as a systematic review of observational studies addressing determinants of the osteoporosis treatment gap and is reported according to PRISMA guidelines (7). The study protocol was registered in PROSPERO (8) on the 3rd of April 2025.

### 2.1 Search strategy and information sources

The electronic search strategy was developed using combinations of MeSH terms and free-text keywords related to osteoporosis and treatment gaps. A representative PubMed (9) search strategy was as follows: (“Osteoporosis”[Mesh] OR osteoporosis[tiab]) AND (“treatment gap”[tiab] OR “care gap”[tiab] OR “treatment disparity”[tiab] OR “under-treatment”[tiab] OR “under-diagnosis”[tiab]). Equivalent search strategies were adapted for Scopus (10), Web of Science (11), ScienceDirect (12), and Europe PMC (13). Searches included studies published up to the 7th of April 2025. Full search strategies for all databases are available from the authors upon reasonable request. Grey literature was not systematically searched; however, reference lists of included studies were screened for additional eligible publications. A total of 1457 references were imported to the Systematic Review Accelerator (SRA) platform (14), where duplicates were removed using the Deduplicator Tool (15). Non-English studies were excluded during initial screening due to feasibility constraints.

### 2.2 Eligibility criteria

Studies were eligible if they met the following criteria:

1. Addressed the osteoporosis treatment gap;
2. Investigated reasons for the treatment gap;
3. Possibly provided an estimate of the treatment gap size.

Adult populations at risk of osteoporosis, with or without prior fragility fractures, were included. “Treatment” was defined as pharmacological therapy for osteoporosis as reported in individual studies. There is no universally accepted definition of the “treatment gap” in osteoporosis. Across studies, it variably refers either to non-treatment following a fragility fracture or to non-treatment among individuals at high fracture risk (based on FRAX, with or without DXA). We therefore did not impose a single definition, but extracted and reported study-specific definitions and interpreted findings within their respective contexts.

### 2.3 Screening and selection process

**Figure 1:**
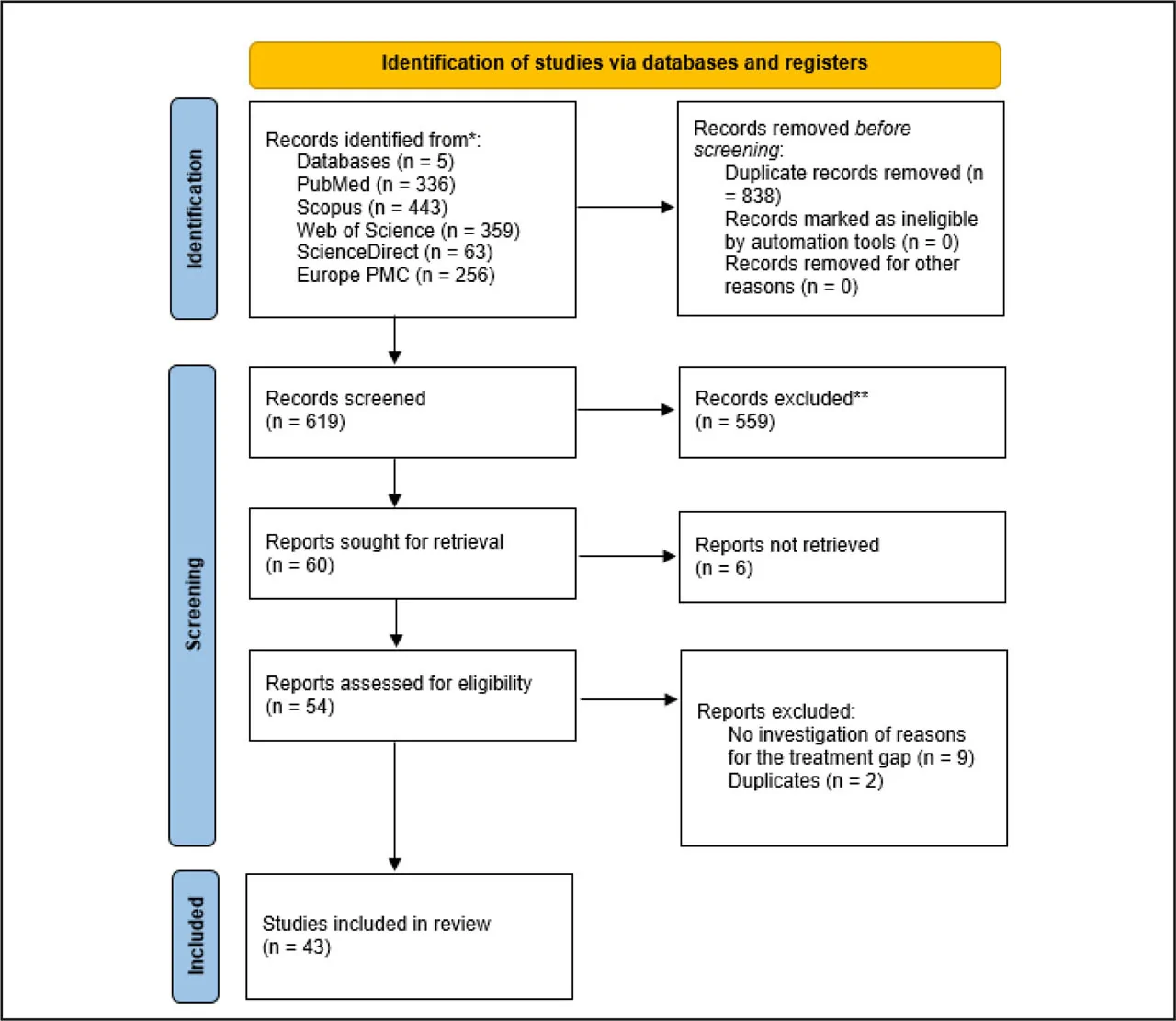
PRISMA 2020 flow diagram (7) representing the selection of 43 (20,21,22,23,24,25,26,27,28,29,30,31,32,33,34,35,36,37,38,39,40,41,42,43,44,45,46,47,48,49,50,51,52,53,54,55,56,57,58,59,60,61,62) original research studies included in the systematic review.

Two authors (K.G.R. and L.P.) independently screened titles and abstracts. Discrepancies were resolved through discussion. Full texts of potentially eligible studies were then retrieved and assessed independently for inclusion by the same reviewers. Non-original research articles and non-English publications were excluded. The selection process was recorded in sufficient detail to complete a PRISMA (7) flow diagram (Figure 1).

### 2.4 Data extraction

A standardised data extraction form was developed and piloted on a subset of included studies. Data were independently extracted by two reviewers (K.G.R. and L.P.) using Microsoft Office 365 Excel (16). Missing data were not sought from the study authors. Given the heterogeneity of included studies, a narrative thematic synthesis was conducted.

### 2.5 Risk of Bias Assessment

Risk of bias (RoB) was assessed using the Newcastle-Ottawa scale for cohort (17) and cross-sectional studies (18) and the Critical Appraisal Skills Programme (CASP) checklist for qualitative studies (19).

## 3 RESULTS

A total of 1,457 records were identified across five databases. After screening, 43 original research studies were included (20,21,22,23,24,25,26,27,28,29,30,31,32,33,34,35,36,37,38,39,40,41,42,43,44,45,46,47,48,49,50,51,52,53,54,55,56,57,58,59,60,61,62). The PRISMA flow diagram is presented in Figure 1 (7).

### 3.1 Study analysis

**Figure 2:**
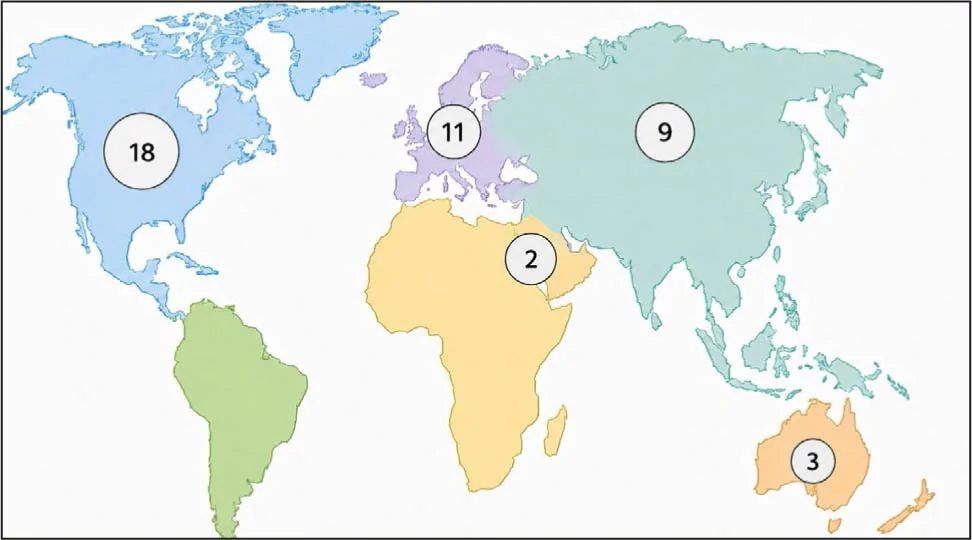
Geographical distribution of included studies (20,21,22,23,24,25,26,27,28,29,30,31,32,33,34,35,36,37,38,39,40,41,42,43,44,45,46,47,48,49,50,51,52,53,54,55,56,57,58,59,60,61,62).

Of the included studies, 5 were prospective cohort studies (20, 29, 37, 41, 43), 22 were retrospective cohort studies (21,22,23,24,25,26,27,28, 30,31,32,33,34,35,36, 38,39,40, 42, 58, 61–62), 15 were cross-sectional studies (44,45,46,47,48,49,50,51,52,53,54,55,56,57, 60), and 1 was a qualitative study (59). 11 studies were conducted in European countries (27,28,29,30,31, 45,46,47,48, 50, 57), 17 in the United States and Canada (24, 33–34, 36,37,38,39,40,41,42,43, 51,52,53, 58, 60–61), 9 in Asian countries (21,22,23, 25–26, 32, 35, 49, 62), 3 in Australia (20, 56, 59), 2 in northern Africa (44, 54) and 1 study combined data from the US, Canada and India (55) (Figure 2). Most studies were conducted in secondary or tertiary care settings (21,22,23,24, 31–32, 37, 41–42, 47, 53) or in mixed healthcare settings (20, 25,26,27, 29–30, 33,34,35,36, 38,39,40, 43–44, 46, 49, 51–52, 54,55,56,57,58,59, 61–62), while five studies were performed exclusively in primary care settings (28, 45, 48, 50, 60). The majority of studies included both male and female participants (20,21,22,23,24,25,26,27,28, 30,31,32, 35,36,37, 39–40, 42, 44,45,46,47, 50,51,52, 58,59,60, 62). Seven studies focused specifically on postmenopausal women (29, 33–34, 38, 41, 48–49), one included only male patients (43), and one examined patients with cancer of both sexes (61). Several studies were survey-based and targeted healthcare professionals (53,54,55,56,57). The summary data from the 43 included studies were organised by outcomes.

### 3.2 Primary outcome

The factors contributing to the treatment gap were categorised (Table 1). Patient-level factors were defined broadly to include both demographic characteristics and clinical or disease-related factors, as these were not consistently distinguished across studies.

**Table 1:** Overview of patient-related (demographic and clinical), physician-related, system-level, and organisational factors associated with variations in osteoporosis treatment rate (20,21,22,23,24,25,26,27,28,29,30,31,32,33,34,35,36,37,38,39,40,41,42,43,44,45,46,47,48,49,50,51,52,53,54,55,56,57,58,59,60,61,62).

**Dimensions**		**Factors contributing to lower treatment rates**	**Factors contributing to higher treatment rates**
**Patient-level factors (demographic and clinical)**	Patient Demographics	Male sex (22, 24,25,26, 30,31,32, 34,35,36,37, 40, 45–46, 50,51,52, 61) Age: in most studies (21, 28–29, 35, 37, 40–41, 46, 49, 50–51, 61), older people are more likely to be treated; some studies show the opposite (26, 31, 38) Body mass index of 30 and over (34, 51) Minority racial and ethnic groups (marginalised groups) (21, 33–34, 39, 50,51,52)	
	Social History	Lower socio-economic status (40, 61) Lower education levels (23) Lower annual income (49) Socioeconomically disadvantaged residential areas (40) Current tobacco use (34)	Married status (49)
	Family History		Family history of osteoporosis or fragility fractures (31, 51)
	Fracture Type	No documented previous fragility fractures (31) Non-vertebral fractures (26–27, 33, 58, 61) Non-major osteoporotic fracture (21, 37)	
	Past Medical History and Investigations	Dementia (22, 33) Diabetes (28, 31, 38) Chronic liver disease (28) Chronic kidney disease (28, 33, 62) Cardiovascular disease and hypertension (28, 38) Arthritis (34) Gastrointestinal troubles (38) Comorbidity (20, 45, 60), high Charlson comorbidity index score (23, 33, 58)	Rheumatoid arthritis and systemic lupus (28, 51) Diagnosis of osteoporosis or bone mineral density testing before a fragility fracture (21, 29, 36, 43–44, 48, 58) Low bone mineral density score (25, 41)
	Pharmacotherapy	Polypharmacy (26, 45, 57) Oestrogen-only hormone replacement therapy (28)	Corticosteroids (28, 51, 58) Prior vitamin D and calcium prescriptions (41)
	Economic Factors	Cost of treatment (49, 54, 60)	
	Patient Belief & Behaviour	Low adherence (47) Refusal of treatment (60) Belief that osteoporosis is not serious (49) More frequent doctor visits (51)	
**Healthcare professionals and healthcare system-level factors**	Physician-related factors	Lack of osteoporosis awareness (54) Limited familiarity with treatment (47, 53) Fear of anti-osteoporotic medications side effects (as perceived barrier by physicians) (54, 60) Workload concerns (53) Differences in prescription practices (46, 61–62) Differences in referral practices (62) Risk assessment not routine practice (44, 47, 55) Failure to investigate (47, 56) Knowledge gaps and lack of confidence (53) Lack of comfort with making treatment changes (60) Intentions to modify the treatment plan (60) Disagreement with recommendations (60), reservations about protocol-based approaches (53) Preference for Fracture Liaison Service (FLS) programmes over standard care (53) Perception by general practitioners that preventive therapy is a specialist's responsibility (57) Interprofessional communication issues and role ambiguity (44, 59) Belief that the current care plan was appropriate (60) Specialist-prescribed treatment plans limiting other providers’ interventions (60)	Both physician and system-related factors: – Transfer to a rehabilitation (42) or a geriatric unit (42) – Preference for FLS (53)
	System/Institutional factors	Lack of patient education and awareness (44, 49, 55, 59) Long intervals between fracture and outpatient visit (25) Length of hospital stay (42) Bone-strengthening or community-based physical activity programmes rare (55)	

### 3.3 Secondary outcome

The treatment gap estimates are presented in Table 2. Because treatment gap estimates were derived from clinically distinct populations, including post-fracture secondary prevention cohorts and broader osteoporosis/high-risk populations, direct comparison of estimates across these categories should be interpreted with caution.

**Table 2:** Country-wise summary of treatment gap estimates in included studies (20,21,22,23,24,25,26,27,28,29,30,31,32,33,34,35,36,37,38,39,40,41, 43,44,45,46,47,48, 51–52, 56, 58, 60,61,62).

**Author & Year**	**Country**	**Treatment Gap Estimate**	Treatment Gap Type*	**Author & Year**	**Country**	**Treatment Gap Estimate**	Treatment Gap Type*
**Feldstein 2005 (58)**	USA	84% (post-hip or vertebral fracture)	Post-fracture secondary prevention	**Leslie 2020 (61)**	Canada	77.1% of cancer patients and 78.2% of non-cancer patients	Post-fracture secondary prevention
**Papaioannou 2008 (43)**	Canada	90%	Post-fracture secondary prevention	**McCloskey 2020 (48)**	Belgium, France, Germany, Ireland, Poland, Slovakia, UK	74.6% (varying from 53% in Ireland to 91% in Germany)	Osteoporosis/high-risk population
**Bessette 2009 (41)**	Canada	84%	Post-fracture secondary prevention	**Skjødt 2020 (30)**	Denmark, Spain (Catalonia), UK	Denmark: 88–90% (2005–2014)	Post-fracture secondary prevention
**Curtis 2009 (52)**	USA	74% even among the highest risk group (Caucasian men with prior fracture)	Osteoporosis/high-risk population	**Malle 2021 (47)**	Austria	82% in women, 92% in men	Post-fracture secondary prevention
**Leslie 2011 (40)**	Canada	93.9% in 1996/1997, 87.7% in 2001/2002, 94.1% in 2007/2008	Post-fracture secondary prevention	**Nakatoh 2021 (26)**	Japan	68.4% (hip fracture), 38.3% (vertebral fracture)	Post-fracture secondary prevention
**Leslie 2012 (39)**	Canada	94.6% in First Nations peoples vs 87.5% in non-Indigenous	Post-fracture secondary prevention	**Papaioannou 2021 (60)**	Canada	63%	Osteoporosis/high-risk population
**Roux 2013 (37)**	Canada	81.2% in the standard care group	Post-fracture secondary prevention	**Skjødt 2021 (27)**	Denmark	85% (2005), 79% (2014)	Post-fracture secondary prevention
**Siris 2013 (38)**	USA	64.3%	Osteoporosis/high-risk population	**Walsh 2021 (28)**	Ireland	79%	Osteoporosis/high-risk population
**Cunningham 2014 (51)**	USA	38.8%	Osteoporosis/high-risk population	**Hoit 2022 (24)**	USA	74% (30 days after operation)	Post-fracture secondary prevention
**Yusuf 2016 (36)**	USA	85.1%	Post-fracture secondary prevention	**Martinez-Laguna 2022 (45)**	Spain	56.8%	Post-fracture secondary prevention
**Gani 2017 (32)**	Singapore	68.5%	Post-fracture secondary prevention	**Wu 2022 (25)**	Taiwan	64.4%	Post-fracture secondary prevention
**Keshishian 2017 (33)**	USA	72.2%	Post-fracture secondary prevention	**Yavropoulou 2022 (46)**	Greece	58%	Osteoporosis/high-risk population
**Mendis 2017 (56)**	Australia	87%	Post-fracture secondary prevention	**Bliuc 2023 (20)**	Australia	78% (women), 86% (men)	Post-fracture secondary prevention
**Sattari 2017 (34)**	USA	78.4%	Osteoporosis/high-risk population	**Cheah 2023 (21)**	Malaysia	82.9%	Post-fracture secondary prevention
**Wang 2017 (35)**	Taiwan	> 60% for women, 90% for men	Osteoporosis/high-risk population	**El Miedany 2023 (44)**	Egypt	82.1%	Post-fracture secondary prevention
**Cheung 2018 (62)**	Hong Kong	From 91% to 85% between 2009 and 2012	Post-fracture secondary prevention	**Fujii 2023 (22)**	Japan	60%	Post-fracture secondary prevention
**Bougioukli 2019 (31)**	Greece	78.6%	Post-fracture secondary prevention	**Mahaisavariya 2023 (23)**	Thailand	68.4%	Post-fracture secondary prevention
**Iconaru 2020 (29)**	Belgium	74.6%–76.5%	Post-fracture secondary prevention				

* Post-fracture secondary prevention refers to treatment eligibility assessed following a fragility fracture. Osteoporosis/high-risk population refers to individuals identified through osteoporosis diagnosis, low bone mineral density, fracture-risk assessment tools (FRAX or CAROC), or population-based estimates of osteoporosis prevalence.

### 3.4 Risk of bias assessment

For cohort studies, NOS scores (17) of 7–9 were considered “good”, 5–6 “fair”, and ≤ 4 “poor”. For cross-sectional studies, adapted NOS scores (18) of 8–10 were considered “good”, 6–7 “fair”, and < 6 “poor”. CASP checklist (19) gives a descriptive rather than a numerical grade. Overall, 32 of 43 studies were rated as “good” quality (21,22,23,24,25,26,27,28,29,30, 33, 35,36,37,38,39,40,41,42, 44,45,46,47,48,49,50,51,52, 56,57,58,59, 61), 8 as “fair” (20, 32, 34, 43, 53, 55, 60), and 4 as “poor” (31, 54, 57, 62). The use of non-validated measurement tools, the lack of sample size justification, and inadequate reporting of response rates limited the two cross-sectional studies, which were rated as poor quality (54, 57). The two cohort studies rated as poor quality (31, 62) were affected by issues including non-representative sampling, failure to ensure absence of the outcome at baseline, reliance on self-reported data, absence of a non-exposed cohort, and insufficient adjustment for confounding factors. Risk of bias assessments informed interpretation, with greater emphasis placed on higher-quality studies. Determinants from studies with a higher risk of bias were generally consistent with those from higher-quality studies; however, all findings were interpreted with caution.

## 4 DISCUSSION

Despite growing awareness of the importance of fracture prevention (63), substantial treatment gaps in osteoporosis care persist globally. These gaps are consistently driven by patient-, physician-, and system-level factors, in line with previous reviews (64–65).

### 4.1 Treatment gap estimates

This systematic review found reported treatment rates ranging from 38.8% to 94.6% across studies. However, comparisons between studies are limited by substantial methodological heterogeneity. To facilitate interpretation, treatment gap estimates were classified according to whether they were derived from post-fracture secondary prevention populations or broader osteoporosis/high-risk populations. Nevertheless, direct comparisons between these categories should be interpreted with caution due to differences in study populations, eligibility criteria, and treatment gap definitions.

There is no uniform definition or approach to calculating the osteoporosis treatment gap. Some studies assessed treatment following fragility fractures as a form of secondary prevention (20,21,22,23,24,25,26,27,28,29,30,31,32, 36–37, 39,40,41, 43, 47, 56, 58, 61–62), while others included broader high-risk populations, including individuals defined by DXA criteria or FRAX-based risk (29). In several studies, the timing of fracture or treatment initiation was unclear (34–35, 38, 44,45,46, 48, 50,51,52).

Despite these differences, similar treatment gaps were observed across clinically distinct groups. For example, one study reported comparable gaps in postmenopausal women defined by T-score ≤ −2.5 and those defined by prior major osteoporotic fracture (29), suggesting that undertreatment is systemic across both diagnosed osteoporosis and high-risk populations.

Overall, variability in definitions and methodologies limits direct comparison and highlights the need for standardised approaches to defining and reporting the treatment gap.

### 4.2 Factors contributing to the treatment gap

The identified determinants were grouped into patient-, physician-, and system-level factors to aid interpretation; this categorisation is descriptive and does not imply causal relationships.

#### 4.2.1 Patient-level factors (demographic and clinical)

Male sex, comorbidity burden, socio-economic disadvantage, and reduced health literacy were consistently associated with lower treatment rates. Male sex was repeatedly linked to lower treatment likelihood despite substantial fracture burden in men (22, 24,25,26, 30,31,32, 34,35,36,37, 40, 45–46, 50,51,52, 61), consistent with evidence that up to 40% of osteoporotic fractures occur in men (66). Age showed a non-linear association with treatment. Most studies reported higher treatment rates with increasing age (21, 28–29, 35, 37, 40–41, 46, 49, 50–51, 61), likely reflecting greater recognition of increased fracture risk. However, very advanced age and multimorbidity were sometimes associated with lower treatment rates (23, 26, 31, 38), suggesting competing clinical priorities and concerns regarding frailty, polypharmacy, and life expectancy. Comorbid conditions, especially dementia, diabetes, cardiovascular and chronic illnesses, were consistently associated with lower treatment rates (20, 22,21,23, 28, 31, 33–34, 38, 45, 60, 62). The same can be said for polypharmacy (26, 45, 57), with physicians often withholding treatment due to concerns over side effects, drug interactions, and life expectancy considerations.

In multiple studies (21, 33–34, 39, 50,51,52), minority and Indigenous populations were consistently less likely to receive treatment compared to white/Caucasian peers, even after a fracture. This disparity highlights structural inequities and cultural or healthcare system barriers in osteoporosis care. Racial and ethnic characteristics are inextricably linked to socio-economic status (SES). Lower SES (40, 61), lower education (23) and lower income (49) were strongly associated with osteoporosis treatment gaps. Cost concerns (49, 54, 60) were explicitly cited by patients as reasons for treatment refusal, especially among lower-income groups. A lower SES is indicative of less frequent and lower-quality care and reduce access to care (67). Additionally, health professionals' implicit bias against patients with low SES has a negative effect on clinical decision-making (68).

Additionally, limited health literacy and awareness of osteoporosis and fracture risk were pervasive across both high- and low-income countries (31, 40, 47, 49, 51). Patient perceptions, attitudes towards osteoporosis, and health behaviours were frequently cited as barriers to assessment and treatment initiation. Malle et al. (47) found that patients lacked an understanding of the indications for treatment or its potential to prevent future fractures. Patients often discontinued medications early due to side effects or a misunderstanding of benefits. Commonly reported issues included a belief that osteoporosis is not a serious condition, particularly in the absence of symptoms or fractures, leading many to decline treatment offers. Roh et al. (49) highlighted that women who believed osteoporosis was a minor health concern or inconvenient to assess were significantly less likely to undergo evaluation or treatment, even when at high risk. A study of postmenopausal women treated with monthly bisphosphonates (69) found that patients' belief in the importance of osteoporosis treatment was a key predictor of adherence.

Clinical severity influenced treatment likelihood. Vertebral and major osteoporotic fractures were more likely to trigger treatment than non-major fractures (21, 26–27, 33, 37, 58, 61). Prior diagnosis of osteoporosis, lower BMD, or prior fractures also increased the likelihood of treatment (21, 25, 29, 31, 36, 41, 43–44, 48, 58).

#### 4.2.2 Physician and system-level factors

Healthcare professionals and system factors emerged as major contributors to the treatment gap. Physician-related barriers were frequently identified, ranging from limited familiarity with osteoporosis management guidelines to concerns about medication side effects, particularly in older or comorbid patients. Beshyah et al. (54) reported that fear of medication adverse effects, workload constraints, and discomfort with protocol-based management approaches were commonly cited reasons. Papaioannou et al. (60) found that many primary physicians intentionally deferred treatment decisions due to perceived appropriateness of current care plans, concerns about intolerance, or uncertainty about modifying specialist-initiated regimens. Interprofessional communication challenges also negatively impacted care continuity. One study (59) highlighted role ambiguity and poor information transfer between system levels, contributing to delayed or missed treatment opportunities. A lack of integrated care pathways and post-fracture assessment services further exacerbated care gaps.

System-level barriers included logistical obstacles such as long waiting times for specialist consultations, limited access to bone density testing, and regional disparities in service provision. Leslie et al. (40) demonstrated marked geographic differences in treatment rates across urban, rural, and remote populations. Furthermore, logistical difficulties, including transportation challenges in older and disabled patients (23) and the absence of community-based fracture prevention programmes (55), compound inequities in access to osteoporosis care.

The observational nature of the included studies limits the interpretation of these determinants. Reported associations do not allow clear separation of confounding from causation, particularly for variables such as age, comorbidity burden, and polypharmacy, which may reflect underlying clinical complexity or competing care priorities rather than independent effects. Furthermore, the distinction between modifiable and non-modifiable factors is not always clear-cut, as observed differences (by sex, age, socio-economic status, or other factors) may reflect broader structural or healthcare system influences. Therefore, these findings should be interpreted cautiously and primarily as indicators of patterns within osteoporosis care rather than direct targets for intervention.

### 4.3 Limitations and strengths

This systematic review has several limitations. First, the review was restricted to English-language publications, which may introduce language bias. This is particularly relevant in a global context, as non-English studies may capture underrepresented healthcare settings and system-level barriers. Consequently, the findings may not fully reflect the global diversity of osteoporosis care and may have limited generalisability. Second, no additional data were sought from the original study authors, which may have limited the completeness of extracted data and the risk of bias assessment. Although the risk of bias was systematically assessed, its impact on the interpretation of findings should be considered. Studies rated as having a higher risk of bias were retained to ensure a comprehensive overview, but were interpreted with caution. Most determinants identified in fair- and poor-quality studies were also reported in studies of good methodological quality, including male sex, ethnicity, comorbidity burden, prior osteoporosis diagnosis or bone mineral density testing, treatment costs, and the lack of routine fracture risk assessment. In contrast, several healthcare professional and organisational factors, including limited familiarity with osteoporosis treatment, knowledge gaps, workload concerns, perceptions regarding responsibility for osteoporosis management, referral and prescription practices, and constraints related to specialist-led treatment plans, were identified primarily in studies with a higher risk of bias (53, 57, 60, 62) and should therefore be interpreted with greater caution. Nevertheless, these factors may represent important hypotheses for future research.

The observational design of included studies, variability in study quality, heterogeneity in outcome definitions, population characteristics and follow-up time limit the strength of inference.

However, the review has notable strengths. It employed a comprehensive search strategy across five major databases, ensuring broad coverage. It includes a diverse, multinational dataset comprising 43 original research studies (20,21,22,23,24,25,26,27,28,29,30,31,32,33,34,35,36,37,38,39,40,41,42,43,44,45,46,47,48,49,50,51,52,53,54,55,56,57,58,59,60,61,62) from a wide range of healthcare systems and populations, thereby improving the findings' generalizability. The categorisation of factors into patient, physician, and system-level domains allowed for a structured analysis of the multifactorial nature of osteoporosis under-treatment. RoB was systematically assessed using validated tools (17,18,19), adding methodological rigour.

### 4.4 Contributions to the field

This review synthesises evidence on gaps in osteoporosis treatment across multiple healthcare systems, providing a comprehensive overview of the determinants of undertreatment. By structuring these into patient-, physician-, and system-level domains, it highlights the multifactorial nature of the problem.

It also demonstrates substantial international variability in post-fracture care, reflecting differences in healthcare organisation, clinical practice, and access to fracture prevention services. Physician-level barriers, such as uncertainty about treatment initiation and inconsistent care pathways, represent actionable targets for intervention. Finally, the review identifies key research gaps, including the optimisation of care for men, improved risk stratification, and the development of scalable post-fracture care models.

## 5 CONCLUSIONS

This systematic review confirms that substantial osteoporosis treatment gaps persist globally, particularly following fragility fractures. These gaps are driven by a combination of patient-, physician-, and system-level factors, including demographic disparities, comorbidity burden, socio-economic disadvantage, and healthcare system limitations.

Marked international variability in post-fracture treatment reflects differences in healthcare organisation, clinical guidelines, and access to diagnostic and treatment services. Physician-related barriers, including concerns about treatment safety, limited confidence in osteoporosis management, and fragmented care pathways, further contribute to undertreatment.

Future research should focus on optimising management for high-risk groups, such as men, improving risk-stratification tools, and developing scalable models of integrated post-fracture care to reduce global disparities in treatment.
